# Connexin Mutants and Cataracts

**DOI:** 10.3389/fphar.2013.00043

**Published:** 2013-04-15

**Authors:** Eric C. Beyer, Lisa Ebihara, Viviana M. Berthoud

**Affiliations:** ^1^Department of Pediatrics, University of ChicagoChicago, IL, USA; ^2^Department of Physiology and Biophysics, Rosalind Franklin Health Sciences UniversityNorth Chicago, IL, USA

**Keywords:** connexin46, connexin50, cataract, lens, gap junction

## Abstract

The lens is a multicellular, but avascular tissue that must stay transparent to allow normal transmission of light and focusing of it on the retina. Damage to lens cells and/or proteins can cause cataracts, opacities that disrupt these processes. The normal survival of the lens is facilitated by an extensive network of gap junctions formed predominantly of connexin46 and connexin50. Mutations of the genes that encode these connexins (*GJA3* and *GJA8*) have been identified and linked to inheritance of cataracts in human families and mouse lines. *In vitro* expression studies of several of these mutants have shown that they exhibit abnormalities that may lead to disease. Many of the mutants reduce or modify intercellular communication due to channel alterations (including loss of function or altered gating) or due to impaired cellular trafficking which reduces the number of gap junction channels within the plasma membrane. However, the abnormalities detected in studies of other mutants suggest that they cause cataracts through other mechanisms including gain of hemichannel function (leading to cell injury and death) and formation of cytoplasmic accumulations (that may act as light scattering particles). These observations and the anticipated results of ongoing studies should elucidate the mechanisms of cataract development due to mutations of lens connexins and abnormalities of other lens proteins. They may also contribute to our understanding of the mechanisms of disease due to connexin mutations in other tissues.

## The Lens and Cataracts

**Figure 1:**
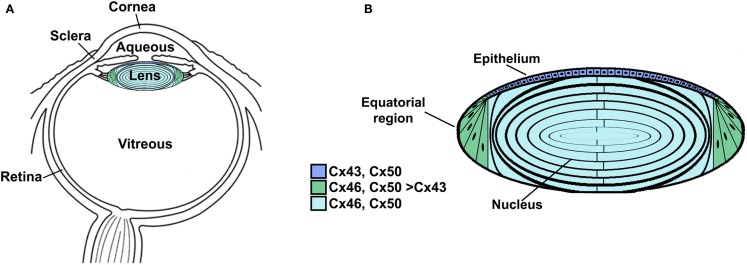
**Anatomy of the eye (A) and structure of the lens (B)**. **(A)** Diagram of the eye illustrating the location of the lens in relation to other structures. The lens is suspended between two transparent fluids, the aqueous and vitreous humors. The aqueous humor fills the space between the cornea and the lens (as well as the posterior chamber). It contains water, oxygen, carbon dioxide, inorganic and organic ions, carbohydrates, glutathione, urea, amino acids, and proteins (including immunoglobulins and growth factors) and is very dynamic (with turnover rates of 1–1.5% per minute) (reviewed by Goel et al., 2010). The aqueous humor stabilizes the ocular structure and contributes to the homeostasis of the avascular structures of the anterior part of the eye by providing nutrition and removing metabolic products. The vitreous is a viscous, gelatinous liquid that fills the space between the lens and the retina. It is essentially a specialized extracellular fluid containing collagen fibers, hyaluronic acid and other soluble proteins, and glycoproteins. The vitreous is rather stagnant, equilibrating very slowly with the plasma. **(B)** Diagram of the lens showing the distribution of connexin isoforms. Cells from the anterior epithelial cell layer express Cx43 and Cx50, differentiating fiber cells express Cx43, Cx46, and Cx50, and fiber cells (including those of the nucleus) contain Cx46 and Cx50.

The lens is a transparent organ whose main function is to transmit light and focus it on the retina. It sits suspended between two clear fluids (the aqueous humor and the vitreous) and has no direct blood supply (Figure 1A). The lens is comprised of two cell types: epithelial cells that form a single layer along the anterior surface and fiber cells that form the bulk of the organ (Figure 1B). At the lens equator, epithelial cells differentiate into fiber cells, a process that involves cell elongation and loss of nuclei and organelles. This differentiation process occurs throughout the lifespan of the organism. Lens fiber cells contain very high concentrations of small soluble proteins called crystallins. These proteins act as chaperones and increase the refractive index (but do not interfere with transparency). Mature fiber cells have limited metabolic activities, are non-dividing, and must survive for the lifespan of the organism. Most of the metabolic, synthetic, and active transport machinery in the lens is localized to surface (nucleated) cells (Mathias and Rae, 2004).

A cataract is a cloudiness or opacity in the lens. It may cause a decrease in vision and may eventually lead to blindness. This disease has substantial public health consequences, because cataracts are the leading cause of blindness worldwide (Resnikoff et al., 2004). Even in those countries where cataractous lenses are surgically removed and replaced with prosthetic intraocular lenses, this eye pathology has a major financial impact. Therefore, many efforts have been devoted to determine the factors that lead to cataract formation and to develop treatments to prevent their formation. Cataracts can be subdivided according to their anatomical location within the lens (e.g., cortical, nuclear, sub-capsular), their appearance (e.g., total, pulverulent), and most commonly by a combination of these two parameters (e.g., nuclear pulverulent). They can also be subdivided according to their etiology (e.g., congenital, disease-related, or age-related). The specific biochemical and structural changes associated with cataract formation are diverse, but a common biochemical change is the presence of high molecular weight insoluble protein aggregates (Moreau and King, 2012).

Because the lens does not have a direct blood supply, the nutrients for the organ all derive from the fluids in which it is suspended. Specifically, the aqueous humor (which is dynamically produced from the plasma and subsequently resorbed) provides the main source for inorganic and organic ions, carbohydrates, glutathione, amino acids, and oxygen. The aqueous humor is also the repository for metabolites and carbon dioxide produced by lens cells. Ions and nutrients reach cells in the interior through an internal “circulation” in which flow of ions and water drives the movement of solutes throughout the organ. A model of this circulation has been developed based on surface currents recorded from lenses (Robinson and Patterson, 1982; Parmelee, 1986; Mathias et al., 1997) and measurements of hydrostatic pressures at different depths within the lens (Gao et al., 2011). In this model, current carried by ions (and associated water and solutes) enters the lens along the extracellular spaces at the anterior and posterior poles, it crosses fiber cell membranes in the lens interior, and it flows back to the surface at the equator (through a cell-to-cell pathway) (Mathias et al., 2007, 2010). The hydrostatic pressure gradient also drives water flow toward the exterior (Gao et al., 2011). The lens circulatory system provides a pathway for internal fiber cells to obtain essential nutrients, remove potentially toxic metabolites, and maintain resting potentials (Goodenough, 1979; Piatigorsky, 1980). Thus, fiber cell survival and the maintenance of transparency depend on the function of epithelial cells and on communication between lens cells (Goodenough, 1992).

## Lens Gap Junctions and Connexins

Intercellular communication among the cells of the lens is facilitated by an extensive network of gap junctions. Gap junctions are membrane specializations that contain clusters of intercellular channels that are permeable to ions and small solutes (≤1 kDa). Lens fiber cells share ions and small metabolites through gap junction channels, and consequently behave as a functional syncytium (Goodenough et al., 1980; Mathias and Rae, 1989). Epithelial and fiber cells contain morphologically and physiologically distinct gap junctions (Rae and Kuszak, 1983; Miller and Goodenough, 1986).

**Figure 2:**
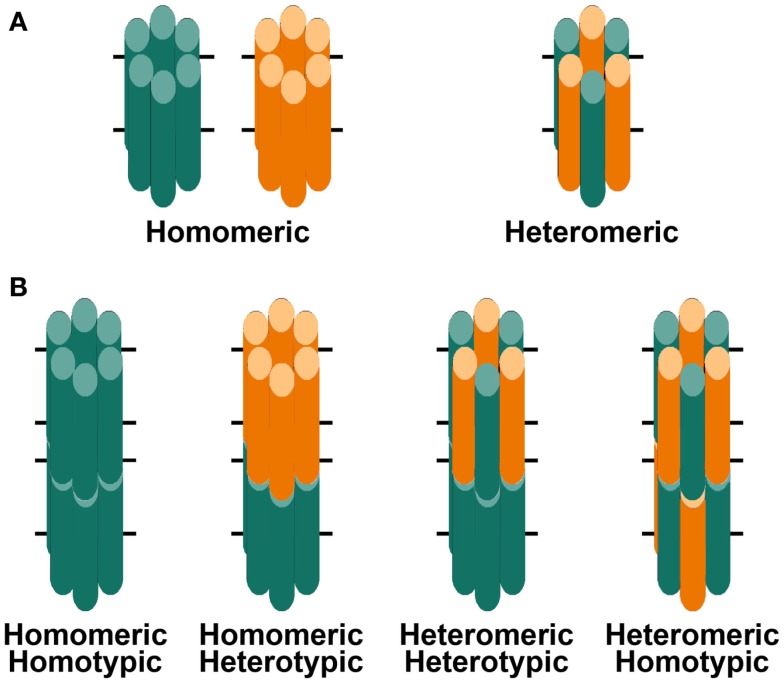
**Diagrams of hemichannels (A) and intercellular channels (B) formed from a single connexin or two different connexins**. **(A)** Six identical connexin subunits (green or orange) can oligomerize to form a homomeric hemichannel. Two co-expressed connexins may oligomerize with each other to form a heteromeric hemichannel. **(B)** Two hemichannels dock with each other to form a complete gap junction channel. Two hemichannels of similar composition form homotypic channels whereas two hemichannels of different composition form heterotypic channels.

Gap junction channels are oligomeric assemblies of members of a family of related proteins called connexins (Cx) (Beyer and Berthoud, 2009). Connexins contain four transmembrane domains connected by two extracellular loops and one cytoplasmic loop. The amino and carboxyl termini of the polypeptide are in the cytoplasm. Six connexins oligomerize to form a connexin hemichannel which traffics to the plasma membrane of one cell where it can dock with another hemichannel from an adjacent cell to form a complete gap junction channel (Figure 2). Channels formed by diverse connexins differ in physiological properties including unitary conductance, permeability, gating, and regulation by different protein kinase-dependent pathways (reviewed in Harris, 2001, 2007; Saez et al., 2003). Thus, the regulation of intercellular communication and the permeation of different molecules in different regions of the lens are determined by the repertoire of connexins expressed.

Three connexins have been identified in the lens with somewhat overlapping expression patterns (Figure 1B). Cx43 is expressed in lens epithelial cells (Musil et al., 1990), but its expression is turned off as epithelial cells in the equatorial region differentiate into fiber cells. Cx50 is also expressed in epithelial cells (TenBroek et al., 1994; Dahm et al., 1999; Rong et al., 2002). Cx46 and Cx50 become abundantly expressed in the differentiating cells and are the two most abundant connexins in lens fiber cells (Paul et al., 1991; White et al., 1992). Cx46 and Cx50 co-localize at gap junction plaques and can form mixed hexamers (Paul et al., 1991; Jiang and Goodenough, 1996). (Heteromeric mixing is illustrated schematically in Figure 2).

Transcripts for a fourth connexin, Cx23, have been detected in the zebrafish embryo lens (Iovine et al., 2008) and in the mouse lens (Puk et al., 2008). Cx23 has been implicated in fiber cell differentiation, because fiber cells in mice expressing a missense mutation of Cx23 did not appear to elongate properly (Puk et al., 2008). Lens cells from other mammalian species may also express Cx23, since it has been identified as an expressed sequence in mRNA from whole eyes or lenses^1^. However, Cx23 transcript was not detected in RNA isolated from human lenses (Sonntag et al., 2009). Moreover, while Cx23 protein has been detected in proteomic studies of mouse lens membrane proteins (Bassnett et al., 2009), it was not detected in human samples (Wang et al., 2013). Therefore, Cx23 is not included in Figure 1B nor considered further in this review.

## Mouse Studies Implicate Connexins in Cataractogenesis

The importance of gap junction-mediated lens intercellular communication for the maintenance of lens transparency has been substantiated by a number of genetic studies in mice. Targeted deletion of either Cx46 or Cx50 results in the development of cataracts in homozygous (but not heterozygous) null mice (Gong et al., 1997; White et al., 1998). The Cx50-null mice have a milder cataract than the Cx46-null mice (Gerido et al., 2003), but Cx50-null mice exhibit microphthalmia and smaller lenses (Gong et al., 1997; White et al., 1998; Rong et al., 2002). The onset of the cataract phenotype in Cx50 knock-out mice occurs within the first postnatal week (White et al., 1998; Rong et al., 2002) whereas the cataracts in Cx46-null mice are visible by the third week of age (Gong et al., 1997). The solubility of some crystallins is decreased in both Cx50- and Cx46-null mice. Double knock-out mice lacking both the Cx46 and Cx50 genes show dense lens opacities that are far more extensive than those observed in either the Cx46 or Cx50 single null mice (Xia et al., 2006).

Transgenic mice over-expressing Cx50 also develop severe cataracts (Chung et al., 2007). This finding suggests that any significant alteration of connexin levels in these cells (either absence or a major increase) may lead to cataract formation.

The role of Cx43 in normal lens function is uncertain. Lenses of prenatal or newborn Cx43-null mice appear normal and transparent ((White et al., 2001) and Berthoud and Beyer, unpublished observations), but Gao and Spray (1998) have observed ultrastructural abnormalities in these lenses. The long-term effects of the loss of Cx43 in the lens cannot be determined in these mice, because global deletion of Cx43 results in neonatal lethality (Reaume et al., 1995). However, the lenses of animals with a conditional deletion of Cx43 are transparent and develop normally through at least 6 months of age, even though intercellular transfer of neurobiotin and Lucifer yellow among epithelial cells is decreased (DeRosa et al., 2009).

The cataract trait in several mutant mouse strains has been mapped to the lens connexin loci. The *No2* mouse carries a missense mutation within the coding region of Cx50 resulting in a change of amino acid residue 47 from aspartate to alanine (Cx50D47A) and develops congenital cataracts (Favor, 1983; Steele et al., 1998); these cataracts are less severe in heterozygous than in homozygous animals. Mice carrying a Cx50 mutation at amino acid residue 64 (changing from valine to alanine, Cx50V64A) exhibit dominantly inherited cataracts (Graw et al., 2001). Another mouse with cataracts, *lop10*, carries a missense mutation at amino acid residue 22 of Cx50 (Cx50G22R) (Chang et al., 2002). Both cataracts and microphthalmia have been observed in mice with the semi-dominant mutation, Cx50R205G (Xia et al., 2012).

## Connexin Mutations and Congenital Cataracts in Humans

**Figure 3:**
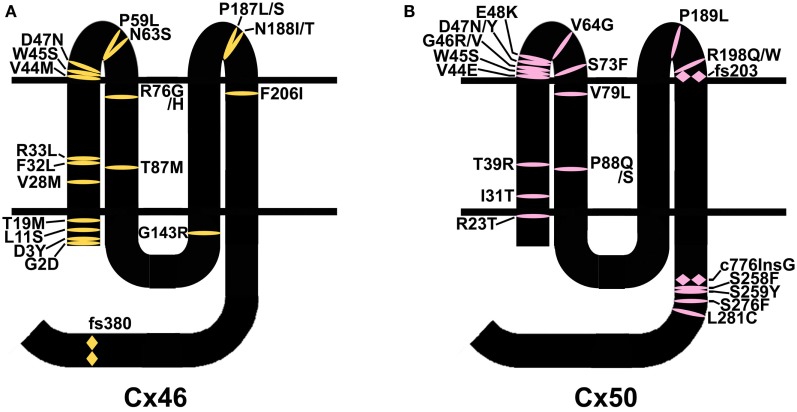
**Diagram illustrating the topology of the human lens connexins, Cx46 (A) and Cx50 (B) and the locations of missense (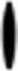) and frame shift (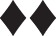) mutations identified in members of families with inherited cataracts**. While included in Table 2, Cx50I247M has not been included in the Cx50 diagram, since it may actually be a polymorphism.

Mutations in lens connexins have been associated with human disease. Missense and frame shift mutations of the Cx46 and Cx50 genes have been identified in members of families with inherited cataracts of various different phenotypes. These mutants and their associated cataract phenotypes are summarized in Table 1 (Cx46) and Table 2 (Cx50). Other than the few exceptions noted in the tables, nearly all of the cataracts are inherited as autosomal dominant traits. The positions of these mutations in relation to the membrane topology of these connexins are shown in Figure 3.

**Table 1:** **Human Cx46 mutants linked to cataract formation**.

Position and alteration	Disease phenotype		Reference
	Cataract appearance	Other features	
G2D	Nuclear pulverulent and posterior polar		Yao et al. (2011)
D3Y	Zonular pulverulent		Addison et al. (2006)
L11S	“Ant-egg”		Hansen et al. (2006)
T19M	Posterior polar		Santhiya et al. (2010)
V28M	Variable		Devi et al. (2005)
F32L	Nuclear pulverulent		Jiang et al. (2003)
R33L	Finely granular embryonal		Guleria et al. (2007a)
V44M			Zhou et al. (2010), Bennett and Shiels (2011)
W45S	Nuclear		Ma et al. (2005)
D47N	Nuclear		Yang et al. (2011)
P59L	Nuclear punctate		Bennett et al. (2004)
N63S	Zonular pulverulent		MacKay et al. (1999)
R76G	Total		Devi et al. (2005)
R76H	Lamellar nuclear opacity with surrounding pulverulent nuclear opacities; lamellar with moderate opacity of the fetal nucleus and Y-shaped condensations in the anterior suture	Dominant inheritance with incomplete penetrance	Burdon et al. (2004), Hansen et al. (2009)
T87M	“Pearl box”		Guleria et al. (2007b)
G143R	Coppock-like		Zhang et al. (2012a)
P187L	Homogeneous zonular pulverulent		Rees et al. (2000)
P187S	Nuclear pulverulent		Ding et al. (2011)
N188I	Nuclear coralliform		Zhang et al. (2012b)
N188T	Nuclear pulverulent		Li et al. (2004)
F206I	Embryonal nuclear		Wang and Zhu (2012)
fs380	Zonular pulverulent		MacKay et al. (1999)

*When the descriptions of the cataract phenotype differed in different reports, both are listed, but separated by a semi-colon. All cataracts were inherited as dominant traits*.

**Table 2:** **Human Cx50 mutants linked to cataract formation**.

Position and alteration	Disease phenotype		Reference
	Cataract appearance	Other features	
R23T	Nuclear		Willoughby et al. (2003)
I31T	Fetal and embryonic nuclear		Wang et al. (2009)
T39R	Complete	Microcornea and iris hypoplasia	Sun et al. (2011)
V44E	Total	Microcornea and variably associated with myopia	Devi and Vijayalakshmi (2006)
W45S	Jellyfish-like		Vanita et al. (2008b)
G46R	Complete	Microcornea	Sun et al. (2011)
G46V	Total		Minogue et al. (2009)
D47N	Nuclear pulverulent		Arora et al. (2008), Wang et al. (2011)
D47Y			Lin et al. (2008)
E48K	“Zonular nuclear” pulverulent		Berry et al. (1999)
V64G	Nuclear		Ma et al. (2005)
S73F	Dense and “star-shaped,” various locations in the nucleus or the poles		Hansen et al. (2009)
V79L	“Full moon” with Y-sutural opacities		Vanita et al. (2006)
P88Q	Lamellar pulverulent; balloon-like with Y-sutural opacities		Arora et al. (2006), Vanita et al. (2008a)
P88S	Zonular pulverulent		Shiels et al. (1998)
P189L	Star-shaped nuclear opacity with a whitish central core	Microcornea	Hansen et al. (2007)
R198Q		Microcornea and variably associated with myopia	Devi and Vijayalakshmi (2006)
R198W		Microcornea without microphthalmia	Hu et al. (2010)
fs203	Total	Recessive inheritance. Associated with nystagmus and amblyopia	Ponnam et al. (2007)
I247M	Zonular pulverulent	May be a polymorphism rather than a disease-causing mutation	Polyakov et al. (2001), Graw et al. (2009)
c776InsG	Triangular cataract	Recessive inheritance	Schmidt et al. (2008)
S258F	Nuclear		Gao et al. (2010)
S259Y			Hansen et al. (2009)
S276F	Pulverulent nuclear		Yan et al. (2008)
L281C	Lamellar/zonular		Kumar et al. (2011)

*When the descriptions of the cataract phenotype differed in different reports, both are listed, but separated by a semi-colon. All cataracts were inherited as dominant traits, except as noted*.

Mutations in Cx43 have been associated with oculodentodigital dysplasia, a disease which is only rarely accompanied by cataracts. Therefore, Cx43 mutations are not considered in this review.

Our laboratories have been interested in determining the biochemical, cell biological and physiological abnormalities in the behaviors of these human mutants. We have expressed different cataract-associated connexin mutants using *in vitro* expression systems, by transfection of communication- and connexin-deficient mammalian cells and by microinjection of *in vitro* transcribed connexin cRNAs into *Xenopus* oocytes. We have identified several different abnormalities (as illustrated by different examples in Table 3). In this paper, we will review some of these findings and consider their implications for understanding cataract pathogenesis. The data summarized will primarily derive from the human connexin mutant experiments performed in our laboratories.

**Table 3:** **Examples of cataract-associated lens connexin mutants with different cellular or physiological abnormalities**.

Mutant	Intercellular channel function	Plaque formation	ER/Golgi localization (without plaques)	Accumulation at other cellular sites	Hemichannel function	Effects on co-expressed wild type connexin	
						Cx50	Cx46
Cx50D47N	None	Absent	Yes	No		No effect	
Cx46fs380	None	Absent	Yes	No	None	No effect	No effect
Cx50R23T	None	Very rare		No		Inhibition	
Cx50P88Q	None	Absent	Yes	Yes		Weak inhibition	
Cx50P88S	None	Absent	No	Yes		Inhibition	
Cx50W45S	None	Normal	No	No	None	Inhibition	
Cx46N63S	None				Reduced and altered sensitivity to divalent cations	No effect	No effect
Cx46D3Y	None	Normal	No	No	Reduced	Hemichannels with altered properties	Hemichannels with altered properties
Cx46L11S	None	Normal	No	No	None	Hemichannels with altered properties	Hemichannels with altered properties
Cx50G46V	Normal	Normal	No	No	Increased	Increased hemichannel function	Increased hemichannel function

*Properties of mutants were all determined for each connexin when expressed by itself, except for the co-expression results. Blanks represent parameters that have not been tested*.

**Figure 4:**
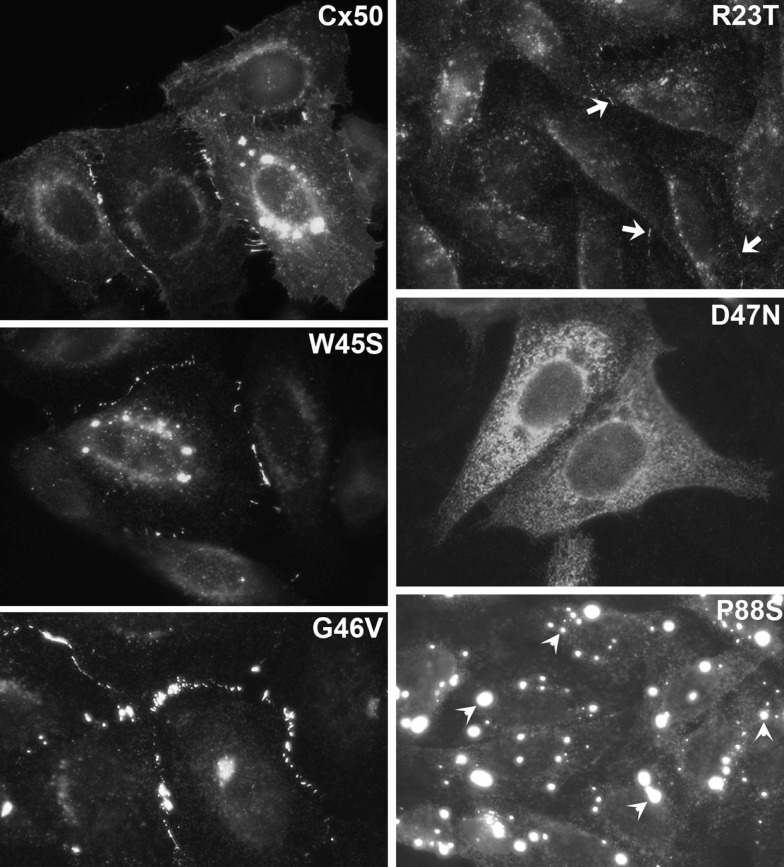
**Immunofluorescent localization of wild type Cx50 and of different cataract-associated Cx50 mutants (R23T, W45S, D47N, G46V, and P88S) after their expression by transfection of HeLa cells**. Similar to wild type Cx50, W45S and G46V show abundant localization in a punctate distribution along appositional membranes corresponding to gap junction plaques. The abundance of plaques is very reduced for R23T, but small spots at appositional membranes are occasionally observed. D47N and P88S show no localization consistent with gap junction plaque formation. D47N is found in a reticular, cytoplasmic distribution. P88S localizes in intensely fluorescent cytoplasmic inclusions. Reproduced and adapted from Berthoud et al. (2003), Arora et al. (2008), Thomas et al. (2008), and Tong et al. (2011).

**Figure 5:**
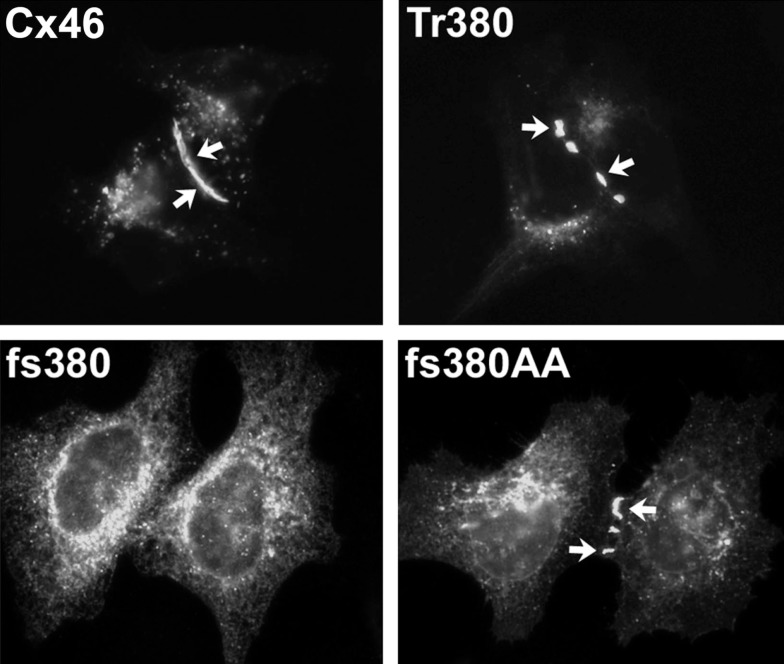
**Immunofluorescent localization of wild type Cx46, the cataract-associated mutant Cx46fs380 (fs380), Cx46 truncated after amino acid 379 (Tr380) and Cx46fs380 with the FF motif replaced by AA (fs380AA) in transfected HeLa cells**. Wild type Cx46 localizes in an intense, linear distribution along appositional membranes as expected for large gap junctions, but such staining is absent for Cx46fs380 which is only found in a cytoplasmic distribution. The cytoplasmic retention must be due to the abnormal sequence in the carboxyl terminus of Cx46fs380, since its removal by truncation (Tr380) restores gap junction formation. Similarly, gap junction formation is restored when the FF motif in Cx46fs380 is replaced with two alanines (fs380AA). Reproduced and adapted from Minogue et al. (2005).

Typically, we have performed functional and cellular screening tests in parallel. These initial studies are designed to test whether a mutant construct induces a level of intercellular conductance above that seen in untransfected cells or water-injected *Xenopus* oocytes and whether the construct leads to the formation of gap junction plaques. Plaque formation is identified as immunoreactive connexin that localizes along appositional membranes with a punctate distribution (examples are shown for wild type Cx46 and Cx50 in Figures 4 and 5).

## Connexin Mutants with Abnormalities of Cellular Biosynthesis or Degradation

The most frequently observed phenotype is a cataract-associated connexin mutant that does not induce a significant intercellular conductance and forms very few or no gap junction plaques. Examples include Cx50R23T, Cx50D47N, Cx50P88S, Cx50P88Q, and Cx46fs380 (Berthoud et al., 2003; Minogue et al., 2005; Arora et al., 2006, 2008; Thomas et al., 2008) (Table 3 and Figures 4 and 5). Among these mutants, Cx50R23T rarely forms small plaques (Thomas et al., 2008), while Cx46fs380 never forms them (Minogue et al., 2005). These differences likely reflect variations in the severity of the trafficking defects and the specific mechanisms involved.

For many of the mutants that do not form plaques, immunoreactive connexin localizes within the cytoplasm. Co-localization studies using antibodies directed against compartments of the protein biosynthetic/secretory pathway have shown that the mutant connexins are contained within the ER, ERGIC, and/or Golgi apparatus (e.g., Cx50D47N and Cx46fs380) (Minogue et al., 2005; Arora et al., 2008). The connexin within these subcellular compartments likely represents mutant protein that has been retained within the export pathway due to misfolding and/or incomplete/improper oligomerization. The interpretation that some of the mutant connexins (e.g., Cx50D47N, Cx50P88Q, Cx50P88S) are misfolded is supported by the presence of gap junction plaques at the plasma membrane after incubation of expressing cells under conditions that should promote protein folding (reduced temperatures or chemical chaperone treatment) (Berthoud et al., 2003; Arora et al., 2006, 2008).

The cytoplasmic retention of a cataract-linked mutant has been explored in detail for Cx46fs380. This mutant contains a frame shift that causes a change in reading frame such that the connexin contains an abnormal C-terminal sequence. We have shown that a two amino acid motif (FF) within the abnormal polypeptide is responsible for its localization within the ERGIC and Golgi (Minogue et al., 2005). This motif has been identified as a trafficking signal in other proteins.

Cx50P88S is an interesting mutant that does not form gap junction plaques when expressed by itself. It has a cytoplasmic localization, but little of the protein is localized within compartments of the biosynthetic/secretory pathway. Rather, this mutant forms cytoplasmic inclusions (1–5/cell) of 0.6–2.7 μm in diameter (Berthoud et al., 2003). Similar inclusions are observed after transfection of several different cell lines. The Cx50P88S accumulations are very long lived (as compared to the turnover of the wild type protein in these expression systems) and correspond to closely apposed circular or semicircular membrane stacks that likely originate from the rough endoplasmic reticulum (Berthoud et al., 2003; Lichtenstein et al., 2009). Some of the Cx50P88S inclusions co-localize with components of the autophagic degradation pathway, and their turnover is altered by interventions that affect autophagy. Thus, the persistence of the Cx50P88S accumulations likely results from insufficient degradation capacity of constitutive autophagy (Lichtenstein et al., 2011). We have also studied another cataract-associated mutant of this connexin at the same position, Cx50P88Q. Although this mutant forms some cytoplasmic inclusions like Cx50P88S, the glutamine substitution seems to have less severe consequences than the serine substitution, since many cells expressing Cx50P88Q contain immunoreactive connexin that co-localizes with markers for the ER or Golgi (Arora et al., 2006).

Additional kinds of cellular and biochemical abnormalities may be predicted for some of the identified cataract-associated mutants. For example, phosphorylation events have been implicated in regulation of many aspects of the connexin life cycle and physiology (reviewed by Solan and Lampe, 2005; Moreno and Lau, 2007). Two Cx50 mutants, Cx50S258F and Cx50S259Y, alter amino acids that are phosphorylated in the wild type protein as shown in a proteomic study of human lens membrane proteins (Wang et al., 2013); however, the cellular and physiological behaviors of these mutants have not yet been studied. In the *Lop10* mouse, expression of the Cx50 mutant reduces the abundance of phosphorylated forms of Cx46, and the Cx46 alterations may contribute to the cataracts in these animals (Chang et al., 2002). The carboxyl termini of both Cx46 and Cx50 are sensitive to cleavage by calpains (Kistler and Bullivant, 1987; Lin et al., 1997; Jacobs et al., 2004). When expressed in heterologous systems, the resulting truncated Cx50 forms channels with reduced function (DeRosa et al., 2006) and sensitivity to intracellular pH (Lin et al., 1998; Xu et al., 2002). It is likely that some of the mutants within the C-terminal region of the connexin may interfere with this cleavage.

These studies allow prediction of some consequences of mutant connexin expression in the lens. All of the mutants with this general phenotype (loss of function due to a severe reduction in the number or complete absence of gap junctions) should reduce intercellular communication between lens fiber cells, regardless of their different mechanisms of retention/accumulation. In the lens, interactions of these mutants with other lens fiber cell proteins (including wild type connexins) might alter their trafficking or function as well. The mutants that are retained within the ER might cause ER stress and might lead to stimulation of the unfolded protein response. A study of the Cx50G22R and Cx50S50P mutant mice concluded that the unfolded protein response might be a contributing factor to the cataracts in these animals (Alapure et al., 2012). The stable cytoplasmic inclusions formed by some mutants (e.g., Cx50P88S) may cause light scattering and act as nucleation particles for accumulation/aggregation of other proteins.

## Connexin Mutants with Abnormalities of Channel Behavior

The gap junction conductance between a pair of cells depends on the number of channels, their open probabilities, and their single channel conductances. The trafficking mutants discussed above are complete (or near complete) loss of function mutants, because they effectively have reduced the number of intercellular channels.

There are other mutants that form non-functional channels (e.g., Cx50W45S, Cx46D3Y, and Cx46L11S) which make abundant gap junction plaques, but have no gap junction channel activity when expressed by themselves (Tong et al., 2011, 2013) (Table 3 and Figure 3). These mutants effectively have an open probability of zero (or no unitary conductance).

Because mutant lens connexins can oligomerize with wild type Cx46 and/or Cx50 to form gap junction channels, several of the cataract-associated mutant lens connexins have been studied for their ability to alter the function of co-expressed wild type lens connexins. Some of these mutants (e.g., Cx46N63S, Cx46fs380) do not inhibit the function of either Cx46 or Cx50 (Pal et al., 2000). Other mutants (e.g., Cx50P88S, Cx50P88Q, Cx50W45S, Cx50E48K, Cx46D3Y, Cx46L11S), decrease the junctional conductance supported by their wild type counterparts (Pal et al., 1999; Arora et al., 2006; Banks et al., 2009; Tong et al., 2011, 2013). In the case of Cx50P88S, a single mutant subunit is sufficient to inhibit function of a full gap junction channel (Pal et al., 1999). Some lens connexin mutants that inhibit their homologous wild type counterparts do not act as strong dominant negative inhibitors of the other lens fiber cell connexin. For example, Cx46D3Y and Cx46L11S inhibit wild type Cx46, but they do not block wild type Cx50 (Tong et al., 2013). In addition, the function of certain lens connexin mutants such as Cx46D3Y is partially rescued by heterotypically pairing cells expressing the mutant connexin with cells expressing wild type lens connexins (Tong et al., 2013). HeLa cells expressing an EGFP fusion protein of Cx46D3Y have been reported to show increased intercellular transfer of Lucifer yellow or ethidium as compared to cells expressing wild type Cx46-EGFP (Schlingmann et al., 2012).

The direct link between loss of connexin function and cataracts is an active area of research. The studies of Matthias and colleagues (reviewed in Mathias et al., 2010) have established that loss of function of either Cx46 or Cx50 (as observed in “knock-out” mice) reduces coupling conductance between lens fiber cells. Reductions of intercellular communication would be anticipated to decrease the circulation of gap junction permeant molecules (including water, ions, and metabolites) between lens cells. Indeed, Gao et al. (2011) observed that reductions in gap junction channels in genetically manipulated mice resulted in a proportional decrease in the gradient of intracellular hydrostatic pressure from the center to the periphery of the lens. One hypothesis links connexin function, calcium levels and proteolysis of crystallins to cataracts based on studies of the Cx46-null mice. These animals have nuclear cataracts associated with generation of a cleaved form of γ-crystallin and significant amounts of NaOH-insoluble α-, β-, and γ-crystallins in the lens nucleus (Gong et al., 1997). Cx46-null mice also have elevated levels of calcium in their lens nuclear regions (Baruch et al., 2001; Gao et al., 2004) and increased activation of calcium-dependent protease activity, likely due to the calpain3 isozyme, Lp82 (Baruch et al., 2001). Moreover, cataract formation is delayed in Cx46-null mice that are also deficient in the gene (*Capn3*) encoding this protease (Tang et al., 2007). However, the mechanism linking Cx46 function to cataracts in humans may be somewhat different, since *CAPN3* mRNA expression has only been identified in skeletal muscle in people (Fougerousse et al., 1998).

Connexin mutants could also affect parameters such as voltage gating, sensitivity to intracellular pH or channel permeability that would lead to altered function, but not necessarily complete loss of function. As summarized in Table 4, Cx46 and Cx50 exhibit some differences in many of these physiological properties. Studies of mouse connexins have identified mutants (like Cx50S50P) that alter the voltage-dependent gating properties of co-expressed Cx46 or Cx50 (DeRosa et al., 2007). Interestingly, mouse Cx50S50P is also a potent inhibitor of co-expressed Cx43, suggesting that intercellular communication may be interrupted in lens epithelial cells where these two connexins are co-expressed (DeRosa et al., 2009). Another mouse mutant, Cx50R205G, blocks the gap junction channel function of co-expressed wild type Cx50, but only affects the gating of Cx46 channels (Xia et al., 2012).

**Table 4:** **Physiological properties of wild type Cx50 and Cx46**.

Property	Cx50	Cx46	Reference
Unitary gap junctional channel conductance	∼220 pS	∼150 pS	Srinivas et al. (1999), Hopperstad et al. (2000)
Ionic selectivity	Cations > anions	Cations > anions	Srinivas et al. (1999), Trexler et al. (2000)
Dye permeability	DAPI, NB ≫ LY	DAPI ≫ LY	Srinivas et al. (1999), Trexler et al. (2000), Thomas et al. (2008)
Sensitivity to transjunctional voltage	++	+	Ebihara and Steiner (1993), White et al. (1994), Srinivas et al. (1999), Hopperstad et al. (2000)
Sensitivity to intracellular pH	+	+	Eckert (2002), Xu et al. (2002)
Sensitivity to activated MAPK	++	−	Shakespeare et al. (2009)
Ability to form functional hemichannels	+	+++	Paul et al. (1991), Ebihara and Steiner (1993), Beahm and Hall (2002)

*Except for the final row, the properties refer to those of intercellular channels formed of each connexin as determined from studies in expression systems*.

*pS, picoSiemens; DAPI, 4′,6-diamidino-2-phenylindole; NB, Neurobiotin; LY, Lucifer yellow; MAPK, Mitogen-activated protein kinase*.

## Connexin Functions Beyond Intercellular Communication

In addition to forming intercellular channels, connexins can also form functional hemichannels that induce large, relatively non-selective conductances in single plasma membranes. These conductances are caused by permeation through “undocked” single connexons. This phenomenon has been best demonstrated in primary cultures of various cells and in expression systems. One of the most dramatic examples is the large permeabilities induced by expression of Cx46 in single *Xenopus* oocytes that can be gated by modulation of extracellular concentrations of divalent cations including calcium (Paul et al., 1991). Because Cx46 hemichannels are mechanosensitive, it has been proposed that their opening plays a physiological role during lens accommodation (Bao et al., 2004). Opening of such hemichannels has been demonstrated in isolated mouse lens fiber cells (Ebihara et al., 2010).The ability of some mutant lens connexins to form functional hemichannels has been assessed. Unlike wild type Cx46, many of the cataract-associated Cx46 mutants do not form functional hemichannels (e.g., Cx46L11S, Cx46fs380). Others exhibit a reduced ability to form them (e.g., Cx46D3Y, Cx46N63S) (Pal et al., 2000; Tong et al., 2013). Thus, entry of sodium and calcium into lens cells expressing these mutants would be impaired if Cx46 hemichannels serve as conduits for these ions in the normal lens.

Cataract-associated mutants may also form hemichannels with altered properties (like gating or charge selectivity) as compared with the wild type connexin. For example, Cx46N63S forms hemichannels with increased sensitivity to the extracellular concentration of magnesium ions (Ebihara et al., 2003). Cx46D3Y forms hemichannels that have altered charge selectivity and voltage gating (Tong et al., 2013).

**Figure 6:**
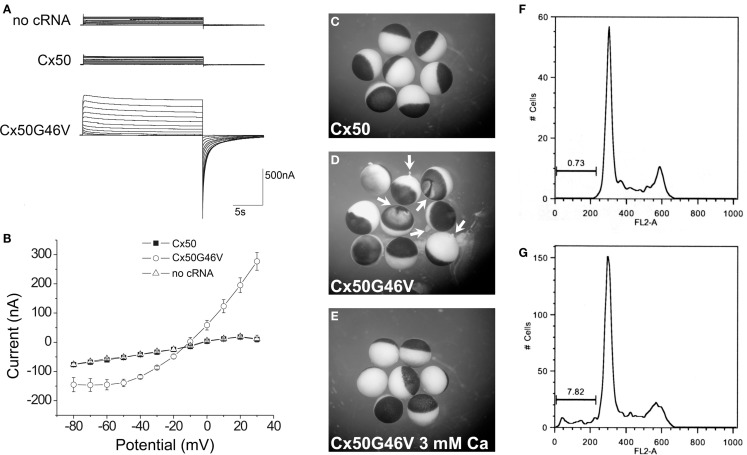
**The Cx50G46V mutant induces large hemichannel currents, cytotoxicity, and apoptosis**. **(A)** Hemichannel currents elicited in response to a series of voltage pulses in control *Xenopus* oocytes (no cRNA) or oocytes injected with cRNA encoding wild type Cx50 or Cx50G46V. The mutant induces much larger currents than wild type Cx50 or control oocytes. **(B)** Steady-state current-voltage relationships in control (no cRNA), wild type Cx50, and Cx50G46V cRNA-injected oocytes. The mutant induces large outward currents that activate on depolarization. **(C–E)** *Xenopus* oocytes were injected with similar amounts of Cx50 (**C**) or Cx50G46V cRNA **(D,E)** and incubated in modified Barth’s solution containing 1 mM Ca^2+^ **(C,D)** or 3 mM Ca^2+^ **(E)** overnight at 18°C. Oocytes injected with Cx50G46V cRNA showed obvious discoloration, membrane disruption, and leakage of yolk (arrows in **D)** when incubated in modified Barth’s solution containing 1 mM Ca^2+^ **(D)** while oocytes injected with Cx50 cRNA showed no apparent detrimental changes when studied under identical conditions **(C)**. The rate of cell death of the Cx50G46V cRNA-injected oocytes was significantly reduced by increasing the external calcium concentration from 1 to 3 mM **(E)**. **(F,G)**. Graphs show the results of cell cycle analysis of propidium iodide-stained HeLa cells induced to express wild type Cx50 **(F)** or Cx50G46V **(G)**. This analysis revealed a substantial population of cells in the sub-G1 fraction, a marker for apoptosis, in cells expressing Cx50G46V but not in cells expressing Cx50 implying that expression of this mutant increased the proportion of apoptotic cells. Reproduced and adapted from Minogue et al. (2009).

A very striking alteration of hemichannel properties is exemplified by Cx50G46V, a mutant found in a patient with total cataract (Minogue et al., 2009). This mutant forms gap junction plaques and supports intercellular communication normally. However, unlike wild type Cx50, Cx50G46V has a greatly increased ability to form functional hemichannels (Minogue et al., 2009; Tong et al., 2011). Expression of this mutant increases the proportion of apoptotic cells and causes cell death (Minogue et al., 2009) (Figure 6), suggesting that opening of the hemichannels would also cause severe cell damage *in vivo*. This cytotoxicity appears dominant, since co-expression of Cx50G46V with wild type Cx46 or Cx50 also decreases cell (oocyte) viability (Tong et al., 2011). Connexin mutants with enhanced hemichannel activity may cause fiber cell death through a complex sequence of events including loss of membrane potential, disruption of transmembrane ion gradients, and entry of calcium ions, leading to activation of intracellular proteases and decreased metabolic activity.

Connexins may also be involved in functions not directly associated with their channel forming ability. Cx50 is required for normal growth of the lens. As mentioned above, mice that are null for Cx50 (but not those null for Cx46) have smaller lenses (Gong et al., 1997; White et al., 1998). The decreased lens size appears to result from reduced proliferation of lens epithelial cells, especially during the first few days of postnatal life (Sellitto et al., 2004). It has been speculated that this proliferative deficiency is due to a connexin function other than intercellular exchange of ions and small molecules, since introduction of Cx46 into the Cx50 locus does not restore normal lens growth to Cx50-null mice (Martinez-Wittinghan et al., 2004). Over-expression of Cx50 also leads to a decrease in lens size (Chung et al., 2007); thus, the proper level of Cx50 expression is required for normal lens development and growth.

The growth and differentiation promoting properties of Cx50 (and the roles of different portions of the molecule) have been further analyzed. Expression of the chicken Cx50 ortholog (including non-functional mutants) or a chicken Cx46 chimera containing the Cx50 carboxyl terminus promotes differentiation of chicken lens cells in culture (Gu et al., 2003; Banks et al., 2007). The mechanism for this “non-channel”-induced effect of Cx50 has not been clarified, but it has been suggested that it involves interactions of its carboxyl terminus with other cellular proteins to modulate intracellular signaling critical for lens cell differentiation. The major intrinsic protein, aquaporin0, is among the lens proteins that can interact with lens connexins (Yu and Jiang, 2004; Yu et al., 2005).

It is plausible that some human Cx50 mutants affect protein interactions or differentiation promoting properties involved in the normal development of the lens and other eye structures. In some of the families with inherited cataracts, affected individuals have small lenses. Moreover, in some pedigrees, the cataracts are associated with additional abnormalities including microcornea, iris hypoplasia, and myopia (see Tables 1 and 2).

## Conclusions and Perspectives

In conclusion, there are many different derangements in the cellular/biochemical behavior and function of the Cx46 and Cx50 mutants that have been linked to cataract formation.

As discussed, the most common abnormality caused by a mutation is improper trafficking and reduced gap junction plaque formation. Such mutations occur at positions throughout the connexin polypeptide, likely because deleterious substitutions of many different residues may cause misfolding (e.g., Cx50D47N). Many identified mutations involve substitutions of amino acids near the amino terminus or within the first transmembrane domain (Figure 3). Since these regions contribute to the channel pore, mutants within them (if targeted properly to the plasma membrane) would be expected to cause functional alterations (e.g., Cx46L11S). The extracellular loops are the other regions with a large number of mutations. Since the structure of these domains is critical for docking between hemichannels and for gating of hemichannels, it would be expected that mutants in this region would affect function if the mutant connexin is appropriately targeted to the plasma membrane (e.g., Cx50G46V).

Elucidating the abnormalities produced by many of these mutations will help to understand the pathogenesis of cataracts and of other diseases caused by mutations in other members of the connexin family. Indeed, reduced formation of gap junctions leading to loss of function is also one of the most commonly observed abnormalities of disease-associated Cx26, Cx32, and Cx43 mutants (reviewed by Laird, 2010; Abrams and Scherer, 2012; Scott et al., 2012; Xu and Nicholson, 2012).

The cataracts associated with Cx46 and Cx50 mutants are predominantly inherited as autosomal dominant traits. This is similar to the inheritance pattern of oculodentodigital dysplasia caused by Cx43 mutants, but differs from Cx32 and most Cx26 mutants which cause disease in a recessive fashion. In some cases, the mutant connexin acts as a “dominant-negative” inhibitor of the function of the co-expressed wild type protein (like Cx50P88S) (Pal et al., 1999). But, other lens connexin mutants show little or no inhibition of the function of the wild type counterparts when studied in expression systems (Table 3). The absence of cataracts in mice that are heterozygous-null for Cx46 or Cx50 (Gong et al., 1997; White et al., 1998) implies that haplodeficiency of these connexins in mice is not sufficient to cause disease. Thus, there must be additional abnormalities conferred by the mutations that lead to lens opacities. Some of these other abnormalities such as formation of cytoplasmic inclusions or gain of hemichannel function have been considered in this review.

It is noteworthy that there are significant differences in the anatomical locations or appearances of the cataracts caused by different lens connexin mutations and among individuals within a single pedigree or in different pedigrees with the same mutation (Tables 1 and 2). Some of the phenotypic dissimilarities between different mutants likely reflect the variety of altered cellular/biochemical mechanisms, since mutants may disrupt different protein–protein interactions and affect different processes. The phenotypic differences among people with the same mutant allele imply that there are other genes that contribute to the phenotype. This conclusion is supported by the strain-dependent differences in severity of the cataracts in Cx46- and Cx50-null mice (Gong et al., 1999; Gerido et al., 2003). The differential severities of the cataracts in Cx46-null mice may partially result from differences in expression of HSP27/25 and ERp29 between strains (Hoehenwarter et al., 2008).

Finally, we can anticipate further progress in the elucidation of the mechanisms involved in cataract formation based on the characterization of additional mutants in expression systems and through the study of corresponding mutants using animal models. Eventually, these studies will contribute to the development of therapeutic approaches to prevent the formation or progression of cataracts due to connexin mutations, and perhaps those due to other genetic or non-genetic causes.

## Conflict of Interest Statement

The authors declare that the research was conducted in the absence of any commercial or financial relationships that could be construed as a potential conflict of interest.

## Footnotes

^1^Accession numbers for *Oryctogalus cuniculus* EST from eye: EB384661.1, EB384662.1; *Bos taurus* EST from lens: EG705869.1; *Macaca mulatta* EST from lens: EG703836.

## References

[B1] Abrams C. K. Scherer S. S. (2012). Gap junctions in inherited human disorders of the central nervous system. Biochim. Biophys. Acta 1818, 2030–2047 10.1016/j.bbamem.2011.08.015 21871435 PMC3771870

[B2] Addison P. K. Berry V. Holden K. R. Espinal D. Rivera B. Su H. (2006). A novel mutation in the connexin 46 gene (*GJA3*) causes autosomal dominant zonular pulverulent cataract in a Hispanic family. Mol. Vis. 12, 791–795 16885921

[B3] Alapure B. V. Stull J. K. Firtina Z. Duncan M. K. (2012). The unfolded protein response is activated in connexin 50 mutant mouse lenses. Exp. Eye Res. 102, 28–37 10.1016/j.exer.2012.06.004 22713599 PMC3461258

[B4] Arora A. Minogue P. J. Liu X. Addison P. K. Russel-Eggitt I. Webster A. R. (2008). A novel connexin50 mutation associated with congenital nuclear pulverulent cataracts. J. Med. Genet. 45, 155–160 10.1136/jmg.2007.051029 18006672 PMC2756454

[B5] Arora A. Minogue P. J. Liu X. Reddy M. A. Ainsworth J. R. Bhattacharya S. S. (2006). A novel *GJA8* mutation is associated with autosomal dominant lamellar pulverulent cataract: further evidence for gap junction dysfunction in human cataract. J. Med. Genet. 43, e2 10.1136/jmg.2005.034108 16397066 PMC2564510

[B6] Banks E. A. Toloue M. M. Shi Q. Zhou Z. J. Liu J. Nicholson B. J. (2009). Connexin mutation that causes dominant congenital cataracts inhibits gap junctions, but not hemichannels, in a dominant negative manner. J. Cell Sci. 122, 378–388 10.1242/jcs.034124 19126675 PMC2650834

[B7] Banks E. A. Yu X. S. Shi Q. Jiang J. X. (2007). Promotion of lens epithelial-fiber differentiation by the C-terminus of connexin 45.6 a role independent of gap junction communication. J. Cell Sci. 120, 3602–3612 10.1242/jcs.000935 17895360

[B8] Bao L. Sachs F. Dahl G. (2004). Connexins are mechanosensitive. Am. J. Physiol. Cell. Physiol. 287, C1389–C1395 10.1152/ajpcell.00220.2004 15475518

[B9] Baruch A. Greenbaum D. Levy E. T. Nielsen P. A. Gilula N. B. Kumar N. M. (2001). Defining a link between gap junction communication, proteolysis, and cataract formation. J Biol. Chem. 276, 28999–29006 10.1074/jbc.M103628200 11395508

[B10] Bassnett S. Wilmarth P. A. David L. L. (2009). The membrane proteome of the mouse lens fiber cell. Mol. Vis. 15, 2448–2463 19956408 PMC2786885

[B11] Beahm D. L. Hall J. E. (2002). Hemichannel and junctional properties of connexin 50. Biophys. J. 82, 2016–2031 10.1016/S0006-3495(02)75550-1 11916859 PMC1301997

[B12] Bennett T. M. MacKay D. S. Knopf H. L. Shiels A. (2004). A novel missense mutation in the gene for gap-junction protein α3 (*GJA3*) associated with autosomal dominant “nuclear punctate” cataracts linked to chromosome 13q. Mol. Vis. 10, 376–382 15208569

[B13] Bennett T. M. Shiels A. (2011). A recurrent missense mutation in *GJA3* associated with autosomal dominant cataract linked to chromosome 13q. Mol. Vis. 17, 2255–2262 21897748 PMC3164684

[B14] Berry V. MacKay D. Khaliq S. Francis P. J. Hameed A. Anwar K. (1999). Connexin 50 mutation in a family with congenital “zonular nuclear” pulverulent cataract of Pakistani origin. Hum. Genet. 105, 168–170 10.1007/s004390051082 10480374

[B15] Berthoud V. M. Minogue P. J. Guo J. Williamson E. K. Xu X. Ebihara L. (2003). Loss of function and impaired degradation of a cataract-associated mutant connexin50. Eur. J. Cell Biol. 82, 209–221 10.1078/0171-9335-00316 12800976 PMC2763359

[B16] Beyer E. C. Berthoud V. M. (2009). “The family of connexin genes,” in Connexins: A Guide, eds Harris A. Locke D. (New York: Humana Press), 3–26

[B17] Burdon K. P. Wirth M. G. Mackey D. A. Russell-Eggitt I. M. Craig J. E. Elder J. E. (2004). A novel mutation in the Connexin 46 gene causes autosomal dominant congenital cataract with incomplete penetrance. J. Med. Genet. 41, e106 10.1136/jmg.2004.018333 15286166 PMC1735867

[B18] Chang B. Wang X. Hawes N. L. Ojakian R. Davisson M. T. Lo W. K. (2002). A *Gja8* (Cx50) point mutation causes an alteration of α3 connexin (Cx46) in semi-dominant cataracts of Lop10 mice. Hum. Mol. Genet. 11, 507–513 10.1093/hmg/11.5.507 11875045

[B19] Chung J. Berthoud V. M. Novak L. Zoltoski R. Heilbrunn B. Minogue P. J. (2007). Transgenic overexpression of connexin50 induces cataracts. Exp. Eye Res. 84, 513–528 10.1016/j.exer.2006.11.004 17217947 PMC1857337

[B20] Dahm R. Van Marle J. Prescott A. R. Quinlan R. A. (1999). Gap junctions containing alpha8-connexin (MP70) in the adult mammalian lens epithelium suggests a re-evaluation of its role in the lens. Exp. Eye Res. 69, 45–56 10.1006/exer.1999.0670 10375448

[B21] DeRosa A. M. Mese G. Li L. Sellitto C. Brink P. R. Gong X. (2009). The cataract causing Cx50-S50P mutant inhibits Cx43 and intercellular communication in the lens epithelium. Exp. Cell Res. 315, 1063–1075 10.1016/j.yexcr.2009.01.017 19331825 PMC2670955

[B22] DeRosa A. M. Mui R. Srinivas M. White T. W. (2006). Functional characterization of a naturally occurring Cx50 truncation. Invest. Ophthalmol. Vis. Sci. 47, 4474–4481 10.1167/iovs.05-1582 17003442 PMC1780262

[B23] DeRosa A. M. Xia C. H. Gong X. White T. W. (2007). The cataract-inducing S50P mutation in Cx50 dominantly alters the channel gating of wild-type lens connexins. J. Cell Sci. 120, 4107–4116 10.1242/jcs.012237 18003700

[B24] Devi R. R. Reena C. Vijayalakshmi P. (2005). Novel mutations in *GJA3* associated with autosomal dominant congenital cataract in the Indian population. Mol. Vis. 11, 846–852 16254549

[B25] Devi R. R. Vijayalakshmi P. (2006). Novel mutations in *GJA8* associated with autosomal dominant congenital cataract and microcornea. Mol. Vis. 12, 190–195 16604058

[B26] Ding X. Wang B. Luo Y. Hu S. Zhou G. Zhou Z. (2011). A novel mutation in the connexin 46 (*GJA3*) gene associated with congenital cataract in a Chinese pedigree. Mol. Vis. 17, 1343–1349 21647269 PMC3107996

[B27] Ebihara L. Liu X. Pal J. D. (2003). Effect of external magnesium and calcium on human connexin46 hemichannels. Biophys. J. 84, 277–286 10.1016/S0006-3495(03)74848-6 12524281 PMC1302609

[B28] Ebihara L. Steiner E. (1993). Properties of a nonjunctional current expressed from a rat connexin46 cDNA in *Xenopus* oocytes. J. Gen. Physiol. 102, 59–74 10.1085/jgp.102.1.59 7690837 PMC2229167

[B29] Ebihara L. Tong J. J. Vertel B. White T. W. Chen T. L. (2010). Properties of connexin 46 hemichannels in dissociated lens fiber cells. Invest. Ophthalmol. Vis. Sci. 52, 882–889 10.1167/iovs.10-6200 20861491 PMC3053112

[B30] Eckert R. (2002). pH gating of lens fibre connexins. Pflugers Arch. 443, 843–851 10.1007/s00424-001-0760-2 11889584

[B31] Favor J. (1983). A comparison of the dominant cataract and recessive specific-locus mutation rates induced by treatment of male mice with ethylnitrosourea. Mutat. Res. 110, 367–382 10.1016/0027-5107(83)90153-7 6877261

[B32] Fougerousse F. Durand M. Suel L. Pourquie O. Delezoide A. L. Romero N. B. (1998). Expression of genes (CAPN3, SGCA, SGCB, and TTN) involved in progressive muscular dystrophies during early human development. Genomics 48, 145–156 10.1006/geno.1997.5160 9521867

[B33] Gao J. Sun X. Martinez-Wittinghan F. J. Gong X. White T. W. Mathias R. T. (2004). Connections between connexins, calcium, and cataracts in the lens. J. Gen. Physiol. 124, 289–300 10.1085/jgp.200409121 15452195 PMC2233908

[B34] Gao J. Sun X. Moore L. C. White T. W. Brink P. R. Mathias R. T. (2011). Lens intracellular hydrostatic pressure is generated by the circulation of sodium and modulated by gap junction coupling. J. Gen. Physiol. 137, 507–520 10.1085/jgp.201010538 21624945 PMC3105514

[B35] Gao X. Cheng J. Lu C. Li X. Li F. Liu C. (2010). A novel mutation in the connexin 50 gene (*GJA8*) associated with autosomal dominant congenital nuclear cataract in a Chinese family. Curr. Eye Res. 35, 597–604 10.3109/02713681003725831 20597646

[B36] Gao Y. Spray D. C. (1998). Structural changes in lenses of mice lacking the gap junction protein connexin43. Invest. Ophthalmol. Vis. Sci. 39, 1198–1209 9620080

[B37] Gerido D. A. Sellitto C. Li L. White T. W. (2003). Genetic background influences cataractogenesis, but not lens growth deficiency, in Cx50-knockout mice. Invest. Ophthalmol. Vis. Sci. 44, 2669–2674 10.1167/iovs.02-1311 12766071

[B38] Goel M. Picciani R. G. Lee R. K. Bhattacharya S. K. (2010). Aqueous humor dynamics: a review. Open. Ophthalmol. J. 4, 52–59 10.2174/1874364101004010052 21293732 PMC3032230

[B39] Gong X. Agopian K. Kumar N. M. Gilula N. B. (1999). Genetic factors influence cataract formation in α_3_ connexin knockout mice. Dev. Genet. 24, 27–32 10.1002/(SICI)1520-6408(1999)24:1/2<27::AID-DVG4>3.0.CO;2-7 10079508

[B40] Gong X. li E. Klier G. Huang Q. Wu Y. Lei H. (1997). Disruption of α_3_ connexin gene leads to proteolysis and cataractogenesis in mice. Cell 91, 833–843 10.1016/S0092-8674(00)80471-7 9413992

[B41] Goodenough D. A. (1979). Lens gap junctions: a structural hypothesis for nonregulated low- resistance intercellular pathways. Invest. Ophthalmol. Vis. Sci. 18, 1104–1122 511455

[B42] Goodenough D. A. (1992). The crystalline lens. A system networked by gap junctional intercellular communication. Semin. Cell Biol. 3, 49–58 10.1016/S1043-4682(10)80007-8 1320431

[B43] Goodenough D. A. Dick J. Lyons J. E. (1980). Lens metabolic cooperation: a study of mouse lens transport and permeability visualized with freeze-substitution autoradiography and electron microscopy. J. Cell Biol. 86, 576–589 10.1083/jcb.86.2.576 6772650 PMC2111473

[B44] Graw J. Loster J. Soewarto D. Fuchs H. Meyer B. Reis A. (2001). Characterization of a mutation in the lens-specific MP70 encoding gene of the mouse leading to a dominant cataract. Exp. Eye Res. 73, 867–876 10.1006/exer.2001.1096 11846517

[B45] Graw J. Schmidt W. Minogue P. J. Rodriguez J. Tong J. J. Klopp N. (2009). The *GJA8* allele encoding CX50I247M is a rare polymorphism, not a cataract-causing mutation. Mol. Vis. 15, 1881–1885 19756179 PMC2743802

[B46] Gu S. Yu X. S. Yin X. Jiang J. X. (2003). Stimulation of lens cell differentiation by gap junction protein connexin 45.6. Invest. Ophthalmol. Vis. Sci. 44, 2103–2111 10.1167/iovs.02-1045 12714649

[B47] Guleria K. Sperling K. Singh D. Varon R. Singh J. R. Vanita V. (2007a). A novel mutation in the connexin 46 (*GJA3*) gene associated with autosomal dominant congenital cataract in an Indian family. Mol. Vis. 13, 1657–1665 17893674

[B48] Guleria K. Vanita V. Singh D. Singh J. R. (2007b). A novel “pearl box” cataract associated with a mutation in the connexin 46 (*GJA3*) gene. Mol. Vis. 13, 797–803 17615540 PMC2768755

[B49] Hansen L. Mikkelsen A. Nurnberg P. Nurnberg G. Anjum I. Eiberg H. (2009). Comprehensive mutational screening in a cohort of Danish families with hereditary congenital cataract. Invest. Ophthalmol. Vis. Sci 50, 3291–3303 10.1167/iovs.08-3149 19182255

[B50] Hansen L. Yao W. Eiberg H. Funding M. Riise R. Kjaer K. W. (2006). The congenital “ant-egg” cataract phenotype is caused by a missense mutation in connexin46. Mol. Vis. 12, 1033–1039 16971895

[B51] Hansen L. Yao W. Eiberg H. Kjaer K. W. Baggesen K. Hejtmancik J. F. (2007). Genetic heterogeneity in microcornea-cataract: five novel mutations in *CRYAA*, *CRYGD*, and *GJA8*. Invest. Ophthalmol. Vis. Sci. 48, 3937–3944 10.1167/iovs.07-0013 17724170

[B52] Harris A. L. (2001). Emerging issues of connexin channels: biophysics fills the gap. Q. Rev. Biophys. 34, 325–472 10.1017/S0033583501003705 11838236

[B53] Harris A. L. (2007). Connexin channel permeability to cytoplasmic molecules. Prog. Biophys. Mol. Biol. 94, 120–143 10.1016/j.pbiomolbio.2007.03.011 17470375 PMC1995164

[B54] Hoehenwarter W. Tang Y. Ackermann R. Pleissner K. P. Schmid M. Stein R. (2008). Identification of proteins that modify cataract of mouse eye lens. Proteomics 8, 5011–5024 10.1002/pmic.200800380 19003866 PMC3018240

[B55] Hopperstad M. G. Srinivas M. Spray D. C. (2000). Properties of gap junction channels formed by Cx46 alone and in combination with Cx50. Biophys. J. 79, 1954–1966 10.1016/S0006-3495(00)76444-7 11023900 PMC1301086

[B56] Hu S. Wang B. Zhou Z. Zhou G. Wang J. Ma X. (2010). A novel mutation in *GJA8* causing congenital cataract-microcornea syndrome in a Chinese pedigree. Mol. Vis. 16, 1585–1592 20806042 PMC2927419

[B57] Iovine M. K. Gumpert A. M. Falk M. M. Mendelson T. C. (2008). Cx23, a connexin with only four extracellular-loop cysteines, forms functional gap junction channels and hemichannels. FEBS Lett. 582, 165–170 10.1016/j.febslet.2007.11.079 18068130 PMC2262847

[B58] Jacobs M. D. Soeller C. Sisley A. M. Cannell M. B. Donaldson P. J. (2004). Gap junction processing and redistribution revealed by quantitative optical measurements of connexin46 epitopes in the lens. Invest. Ophthalmol. Vis. Sci. 45, 191–199 10.1167/iovs.03-0148 14691173

[B59] Jiang H. Jin Y. Bu L. Zhang W. Liu J. Cui B. (2003). A novel mutation in *GJA3* (connexin46) for autosomal dominant congenital nuclear pulverulent cataract. Mol. Vis. 9, 579–583 14627959

[B60] Jiang J. X. Goodenough D. A. (1996). Heteromeric connexons in lens gap junction channels. Proc. Natl. Acad. Sci. U.S.A. 93, 1287–1291 10.1073/pnas.93.20.11236 8577756 PMC40072

[B61] Kistler J. Bullivant S. (1987). Protein processing in lens intercellular junctions: cleavage of MP70 to MP38. Invest. Ophthalmol. Vis. Sci. 28, 1687–1692 3654141

[B62] Kumar M. Agarwal T. Khokhar S. Kumar M. Kaur P. Roy T. S. (2011). Mutation screening and genotype phenotype correlation of α-crystallin, γ-crystallin and GJA8 gene in congenital cataract. Mol. Vis. 17, 693–707 21423869 PMC3060158

[B63] Laird D. W. (2010). The gap junction proteome and its relationship to disease. Trends Cell Biol. 20, 92–101 10.1016/j.tcb.2009.11.001 19944606

[B64] Li Y. Wang J. Dong B. Man H. (2004). A novel connexin46 (*GJA3*) mutation in autosomal dominant congenital nuclear pulverulent cataract. Mol. Vis. 10, 668–671 15448617

[B65] Lichtenstein A. Gaietta G. M. Deerinck T. J. Crum J. Sosinsky G. E. Beyer E. C. (2009). The cytoplasmic accumulations of the cataract-associated mutant, Connexin50P88S, are long-lived and form in the endoplasmic reticulum. Exp. Eye Res. 88, 600–609 10.1016/j.exer.2008.11.024 19073179 PMC2695785

[B66] Lichtenstein A. Minogue P. J. Beyer E. C. Berthoud V. M. (2011). Autophagy: a pathway that contributes to connexin degradation. J. Cell Sci. 124, 910–920 10.1242/jcs.073072 21378309 PMC3048889

[B67] Lin J. S. Eckert R. Kistler J. Donaldson P. (1998). Spatial differences in gap junction gating in the lens are a consequence of connexin cleavage. Eur. J. Cell Biol. 76, 246–250 10.1016/S0171-9335(98)80002-2 9765054

[B68] Lin J. S. Fitzgerald S. Dong Y. Knight C. Donaldson P. Kistler J. (1997). Processing of the gap junction protein connexin50 in the ocular lens is accomplished by calpain. Eur. J. Cell Biol. 73, 141–149 10.1016/S0006-3495(97)78055-X 9208227

[B69] Lin Y. Liu N. N. Lei C. T. Fan Y. C. Liu X. Q. Yang Y. (2008). [A novel GJA8 mutation in a Chinese family with autosomal dominant congenital cataract]. Zhonghua Yi. Xue. Yi. Chuan Xue. Za Zhi. 25, 59–62 18247306

[B70] Ma Z. Zheng J. Yang F. Ji J. Li X. Tang X. (2005). Two novel mutations of connexin genes in Chinese families with autosomal dominant congenital nuclear cataract. Br. J Ophthalmol. 89, 1535–1537 10.1136/bjo.2005.075184 16234473 PMC1772944

[B71] MacKay D. Ionides A. Kibar Z. Rouleau G. Berry V. Moore A. (1999). Connexin46 mutations in autosomal dominant congenital cataract. Am. J. Hum. Genet. 64, 1357–1364 10.1086/302383 10205266 PMC1377871

[B72] Martinez-Wittinghan F. J. Sellitto C. White T. W. Mathias R. T. Paul D. Goodenough D. A. (2004). Lens gap junctional coupling is modulated by connexin identity and the locus of gene expression. Invest. Ophthalmol. Vis. Sci. 45, 3629–3637 10.1167/iovs.04-0445 15452070

[B73] Mathias R. T. Kistler J. Donaldson P. (2007). The lens circulation. J. Membr. Biol. 216, 1–16 10.1007/s00232-007-9019-y 17568975

[B74] Mathias R. T. Rae J. L. (1989). “Cell to cell communication in lens,” in Cell Interaction and Gap Junction, eds Sperelakis N. Cole W. C. (Boca Raton, FL: CRC Press, Inc.), 29–50

[B75] Mathias R. T. Rae J. L. (2004). The lens: local transport and global transparency. Exp. Eye Res 78, 689–698 10.1016/j.exer.2003.07.001 15106948

[B76] Mathias R. T. Rae J. L. Baldo G. J. (1997). Physiological properties of the normal lens. Physiol. Rev. 77, 21–50 9016299 10.1152/physrev.1997.77.1.21

[B77] Mathias R. T. White T. W. Gong X. (2010). Lens gap junctions in growth, differentiation, and homeostasis. Physiol. Rev. 90, 179–206 10.1152/physrev.00034.2009 20086076 PMC4627646

[B78] Miller T. M. Goodenough D. A. (1986). Evidence for two physiologically distinct gap junctions expressed by the chick lens epithelial cell. J. Cell Biol. 102, 194–199 10.1083/jcb.102.1.194 3079768 PMC2114033

[B79] Minogue P. J. Liu X. Ebihara L. Beyer E. C. Berthoud V. M. (2005). An aberrant sequence in a connexin46 mutant underlies congenital cataracts. J. Biol. Chem. 280, 40788–40795 10.1074/jbc.M504765200 16204255 PMC2720622

[B80] Minogue P. J. Tong J. J. Arora A. Russell-Eggitt I. Hunt D. M. Moore A. T. (2009). A mutant connexin50 with enhanced hemichannel function leads to cell death. Invest. Ophthalmol. Vis. Sci. 50, 5837–5845 10.1167/iovs.09-3759 19684000 PMC2788668

[B81] Moreau K. L. King J. A. (2012). Protein misfolding and aggregation in cataract disease and prospects for prevention. Tr. Molec. Med. 18, 273–282 10.1016/j.molmed.2012.03.005 PMC3621977 22520268

[B82] Moreno A. P. Lau A. F. (2007). Gap junction channel gating modulated through protein phosphorylation. Prog. Biophys. Mol. Biol. 94, 107–119 10.1016/j.pbiomolbio.2007.03.004 17507079 PMC1973155

[B83] Musil L. S. Beyer E. C. Goodenough D. A. (1990). Expression of the gap junction protein connexin43 in embryonic chick lens: molecular cloning, ultrastructural localization, and post-translational phosphorylation. J. Membr. Biol. 116, 163–175 10.1007/BF01868674 2166164

[B84] Pal J. D. Berthoud V. M. Beyer E. C. MacKay D. Shiels A. Ebihara L. (1999). Molecular mechanism underlying a Cx50-linked congenital cataract. Am. J. Physiol. Cell. Physiol. 276, C1443–C1446 10.1152/ajpcell.1999.276.6.C1443 10362609

[B85] Pal J. D. Liu X. MacKay D. Shiels A. Berthoud V. M. Beyer E. C. (2000). Connexin46 mutations linked to congenital cataract show loss of gap junction channel function. Am. J. Physiol. Cell. Physiol. 279, C596–C602 10942709 10.1152/ajpcell.2000.279.3.C596

[B86] Parmelee J. T. (1986). Measurement of steady currents around the frog lens. Exp. Eye Res. 42, 433–441 10.1016/0014-4835(86)90003-5 3487463

[B87] Paul D. L. Ebihara L. Takemoto L. J. Swenson K. I. Goodenough D. A. (1991). Connexin46, a novel lens gap junction protein, induces voltage- gated currents in nonjunctional plasma membrane of Xenopus oocytes. J. Cell Biol. 115, 1077–1089 10.1083/jcb.115.4.1077 1659572 PMC2289939

[B88] Piatigorsky J. (1980). Intracellular ions, protein metabolism, and cataract formation. Curr. Top. Eye Res. 3, 1–39 7047087

[B89] Polyakov A. V. Shagina I. A. Khlebnikova O. V. Evgrafov O. V. (2001). Mutation in the connexin 50 gene (*GJA8*) in a Russian family with zonular pulverulent cataract. Clin. Genet. 60, 476–478 10.1034/j.1399-0004.2001.600614.x 11846744

[B90] Ponnam S. P. Ramesha K. Tejwani S. Ramamurthy B. Kannabiran C. (2007). Mutation of the gap junction protein alpha 8 (*GJA8*) gene causes autosomal recessive cataract. J. Med. Genet. 44, e85 10.1136/jmg.2007.050138 17601931 PMC2598012

[B91] Puk O. Loster J. Dalke C. Soewarto D. Fuchs H. Budde B. (2008). Mutation in a novel connexin-like gene (*Gjf1*) in the mouse affects early lens development and causes a variable small-eye phenotype. Invest. Ophthalmol. Vis. Sci. 49, 1525–1532 10.1167/iovs.07-1033 18385072

[B92] Rae J. L. Kuszak J. R. (1983). The electrical coupling of epithelium and fibers in the frog lens. Exp. Eye Res. 36, 317 10.1016/0014-4835(83)90114-8 6601018

[B93] Reaume A. G. Desousa P. A. Kulkarni S. Langille B. L. Zhu D. G. Davies T. C. (1995). Cardiac malformation in neonatal mice lacking connexin43. Science 267, 1831–1834 10.1126/science.7892609 7892609

[B94] Rees M. I. Watts P. Fenton I. Clarke A. Snell R. G. Owen M. J. (2000). Further evidence of autosomal dominant congenital zonular pulverulent cataracts linked to 13q11 (CZP3) and a novel mutation in connexin 46 (GJA3). Hum. Genet. 106, 206–209 10.1007/s004390051029 10746562

[B95] Resnikoff S. Pascolini D. Etya’ale D. Kocur I. Pararajasegaram R. Pokharel G. P. (2004). Global data on visual impairment in the year 2002. Bull. World Health Organ. 82, 844–851 15640920 PMC2623053

[B96] Robinson K. R. Patterson J. W. (1982). Localization of steady currents in the lens. Curr. Eye Res. 2, 843–847 10.3109/02713688209020020 7187641

[B97] Rong P. Wang X. Niesman I. Wu Y. Benedetti L. E. Dunia I. (2002). Disruption of *Gja8* (α8 connexin) in mice leads to microphthalmia associated with retardation of lens growth and lens fiber maturation. Development 129, 167–174 11782410 10.1242/dev.129.1.167

[B98] Saez J. C. Berthoud V. M. Branes M. C. Martinez A. D. Beyer E. C. (2003). Plasma membrane channels formed by connexins: their regulation and functions. Physiol. Rev. 83, 1359–1400 14506308 10.1152/physrev.00007.2003

[B99] Santhiya S. T. Kumar G. S. Sudhakar P. Gupta N. Klopp N. Illig T. (2010). Molecular analysis of cataract families in India: new mutations in the *CRYBB2* and *GJA3* genes and rare polymorphisms. Mol. Vis. 16, 1837–1847 21031021 PMC2956670

[B100] Schlingmann B. Schadzek P. Busko S. Heisterkamp A. Ngezahayo A. (2012). Cataract-associated D3Y mutation of human connexin46 (hCx46) increases the dye coupling of gap junction channels and suppresses the voltage sensitivity of hemichannels. J. Bioenerg. Biomembr. 44, 607–614 10.1007/s10863-012-9461-0 22843197

[B101] Schmidt W. Klopp N. Illig T. Graw J. (2008). A novel *GJA8* mutation causing a recessive triangular cataract. Mol. Vis. 14, 851–856 18483562 PMC2375854

[B102] Scott C. A. Tattersall D. O’Toole E. A. Kelsell D. P. (2012). Connexins in epidermal homeostasis and skin disease. Biochim. Biophys. Acta 1818, 1952–1961 10.1016/j.bbamem.2011.09.004 21933662

[B103] Sellitto C. Li L. White T. W. (2004). Connexin50 is essential for normal postnatal lens cell proliferation. Invest. Ophthalmol. Vis. Sci. 45, 3196–3202 10.1167/iovs.04-0194 15326140

[B104] Shakespeare T. I. Sellitto C. Li L. Rubinos C. Gong X. Srinivas M. (2009). Interaction between Connexin50 and mitogen-activated protein kinase signaling in lens homeostasis. Mol. Biol. Cell 20, 2582–2592 10.1091/mbc.E08-12-1257 19321662 PMC2682599

[B105] Shiels A. MacKay D. Ionides A. Berry V. Moore A. Bhattacharya S. (1998). A missense mutation in the human connexin50 gene (*GJA8*) underlies autosomal dominant “zonular pulverulent” cataract, on chromosome 1q. Am. J. Hum. Genet. 62, 526–532 10.1086/301762 9497259 PMC1376956

[B106] Solan J. L. Lampe P. D. (2005). Connexin phosphorylation as a regulatory event linked to gap junction channel assembly. Biochim. Biophys. Acta 1711, 154–163 10.1016/j.bbamem.2004.09.013 15955300

[B107] Sonntag S. Sohl G. Dobrowolski R. Zhang J. Theis M. Winterhager E. (2009). Mouse lens connexin23 (Gje1) does not form functional gap junction channels but causes enhanced ATP release from HeLa cells. Eur. J. Cell Biol. 88, 65–77 10.1016/j.ejcb.2008.08.004 18849090 PMC2719720

[B108] Srinivas M. Costa M. Gao Y. Fort A. Fishman G. I. Spray D. C. (1999). Voltage dependence of macroscopic and unitary currents of gap junction channels formed by mouse connexin50 expressed in rat neuroblastoma cells. J. Physiol. (Lond.) 517(Pt 3), 673–689 10.1111/j.1469-7793.1999.0673s.x 10358109 PMC2269370

[B109] Sun W. Xiao X. Li S. Guo X. Zhang Q. (2011). Mutational screening of six genes in Chinese patients with congenital cataract and microcornea. Mol. Vis. 17, 1508–1513 21686328 PMC3115747

[B110] Steele E. C. J. Lyon M. F. Favor J. Guillot P. V. Boyd Y. Church R. L. (1998). A mutation in the connexin 50 (Cx50) gene is a candidate for the No2 mouse cataract. Curr. Eye Res. 17, 883–889 10.1076/ceyr.17.9.883.5144 9746435

[B111] Tang Y. Liu X. Zoltoski R. K. Novak L. A. Herrera R. A. Richard I. (2007). Age-related cataracts in α3Cx46-knockout mice are dependent on a calpain 3 isoform. Invest. Ophthalmol. Vis. Sci 48, 2685–2694 10.1167/iovs.06-0926 17525200 PMC1959511

[B112] TenBroek E. M. Johnson R. Louis C. F. (1994). Cell-to-cell communication in a differentiating ovine lens culture system. Invest. Ophthalmol. Vis. Sci. 35, 215–228 8300349

[B113] Thomas B. C. Minogue P. J. Valiunas V. Kanaporis G. Brink P. R. Berthoud V. M. (2008). Cataracts are caused by alterations of a critical N-terminal positive charge in connexin50. Invest. Ophthalmol. Vis. Sci. 49, 2549–2556 10.1167/iovs.07-1658 18326694 PMC2694449

[B114] Tong J. J. Minogue P. J. Guo W. Chen T. L. Beyer E. C. Berthoud V. M. (2011). Different consequences of cataract-associated mutations at adjacent positions in the first extracellular boundary of connexin50. Am. J. Physiol. Cell. Physiol. 300, C1055–C1064 10.1152/ajpcell.00384.2010 21228318 PMC3093948

[B115] Tong J. J. Sohn B. C. Lam A. Walters D. E. Vertel B. M. Ebihara L. (2013). Properties of two cataract associated mutations located in the N-terminus of Connexin 46. Am. J. Physiol Cell Physiol. 10.1152/apjcell.00344.2012 PMC3651606 23302783

[B116] Trexler E. B. Bukauskas F. F. Kronengold J. Bargiello T. A. Verselis V. K. (2000). The first extracellular loop domain is a major determinant of charge selectivity in connexin46 channels. Biophys. J. 79, 3036–3051 10.1016/S0006-3495(00)76539-8 11106610 PMC1301181

[B117] Vanita V. Hennies H. C. Singh D. Nurnberg P. Sperling K. Singh J. R. (2006). A novel mutation in *GJA8* associated with autosomal dominant congenital cataract in a family of Indian origin. Mol. Vis. 12, 1217–1222 17110920

[B118] Vanita V. Singh J. R. Singh D. Varon R. Sperling K. (2008a). A mutation in *GJA8* (p.P88Q) is associated with “balloon-like” cataract with Y-sutural opacities in a family of Indian origin. Mol. Vis. 14, 1171–1175 18587493 PMC2435161

[B119] Vanita V. Singh J. R. Singh D. Varon R. Sperling K. (2008b). A novel mutation in *GJA8* associated with jellyfish-like cataract in a family of Indian origin. Mol. Vis. 14, 323–326 18334946 PMC2255026

[B120] Wang K. Wang B. Wang J. Zhou S. Yun B. Suo P. (2009). A novel GJA8 mutation (p.I31T) causing autosomal dominant congenital cataract in a Chinese family. Mol. Vis. 15, 2813–2820 20019893 PMC2794658

[B121] Wang K. J. Zhu S. Q. (2012). A novel p.F206I mutation in Cx46 associated with autosomal dominant congenital cataract. Mol. Vis. 18, 968–973 22550389 PMC3339038

[B122] Wang L. Luo Y. Wen W. Zhang S. Lu Y. (2011). Another evidence for a D47N mutation in *GJA8* associated with autosomal dominant congenital cataract. Mol. Vis. 17, 2380–2385 21921990 PMC3171490

[B123] Wang Z. Han J. David L. L. Schey K. L. (2013). Proteomics and phosphoproteomics analysis of human lens fiber cell membranes. Invest. Ophthalmol. Vis. Sci. 54, 1135–1143 10.1167/iovs.12-10468 23349431 PMC3567755

[B124] White T. W. Bruzzone R. Goodenough D. A. Paul D. L. (1992). Mouse Cx50, a functional member of the connexin family of gap junction proteins, is the lens fiber protein MP70. Mol. Biol. Cell 3, 711–720 1325220 10.1091/mbc.3.7.711 PMC275629

[B125] White T. W. Bruzzone R. Wolfram S. Paul D. L. Goodenough D. A. (1994). Selective interactions among the multiple connexin proteins expressed in the vertegrate lens: the second extracellular domain is a determinant of compatibility between connexins. J. Cell Biol. 125, 879–892 10.1083/jcb.125.4.879 8188753 PMC2120075

[B126] White T. W. Goodenough D. A. Paul D. L. (1998). Targeted ablation of connexin50 in mice results in microphthalmia and zonlular pulverulent cataracts. J. Cell Biol. 143, 815–825 10.1083/jcb.143.3.815 9813099 PMC2148149

[B127] White T. W. Sellitto C. Paul D. L. Goodenough D. A. (2001). Prenatal lens development in connexin43 and connexin50 double knockout mice. Invest. Ophthalmol. Vis. Sci. 42, 2916–2923 11687537

[B128] Willoughby C. E. Arab S. Gandhi R. Zeinali S. Arab S. Luk D. (2003). A novel *GJA8* mutation in an Iranian family with progressive autosomal dominant congenital nuclear cataract. J. Med. Genet. 40, e124 10.1136/jmg.40.11.e124 14627691 PMC1735309

[B129] Xia C. H. Chang B. DeRosa A. M. Cheng C. White T. W. Gong X. (2012). Cataracts and microphthalmia caused by a gja8 mutation in extracellular loop 2. PLoS ONE. 7:e52894 10.1371/journal.pone.0052894 23300808 PMC3530494

[B130] Xia C. H. Cheng C. Huang Q. Cheung D. Li L. Dunia I. (2006). Absence of α3 (Cx46) and α8 (Cx50) connexins leads to cataracts by affecting lens inner fiber cells. Exp. Eye Res. 83, 688–696 10.1016/j.exer.2006.03.013 16696970

[B131] Xu J. Nicholson B. J. (2012). The role of connexins in ear and skin physiology – functional insights from disease-associated mutations. Biochim. Biophys. Acta 1828, 167–178 22796187 10.1016/j.bbamem.2012.06.024 PMC3521577

[B132] Xu X. Berthoud V. M. Beyer E. C. Ebihara L. (2002). Functional role of the carboxyl terminal domain of human connexin 50 in gap junctional channels. J. Membr. Biol. 186, 101–112 10.1007/s00232-001-0139-5 11944087 PMC2744361

[B133] Yan M. Xiong C. Ye S. Q. Chen Y. Ke M. Zheng F. (2008). A novel connexin 50 (*GJA8*) mutation in a Chinese family with a dominant congenital pulverulent nuclear cataract. Mol. Vis. 14, 418–424 18334966 PMC2268715

[B134] Yang G. Xing B. Liu G. Lu X. Jia X. Lu X. (2011). A novel mutation in the *GJA3* (connexin46) gene is associated with autosomal dominant congenital nuclear cataract in a Chinese family. Mol. Vis. 17, 1070–1073 21552498 PMC3086624

[B135] Yao K. Wang W. Zhu Y. Jin C. Shentu X. Jiang J. (2011). A novel *GJA3* mutation associated with congenital nuclear pulverulent and posterior polar cataract in a Chinese family. Hum. Mutat. 32, 1367–1370 10.1002/humu.21552 21681855

[B136] Yu X. S. Jiang J. X. (2004). Interaction of major intrinsic protein (aquaporin-0) with fiber connexins in lens development. J. Cell Sci. 117, 871–880 10.1242/jcs.01239 14762116

[B137] Yu X. S. Yin X. Lafer E. M. Jiang J. X. (2005). Developmental regulation of the direct interaction between the intracellular loop of connexin 45.6 and the C terminus of major intrinsic protein (aquaporin-0). J. Biol. Chem. 280, 22081–22090 10.1074/jbc.M508138200 15802270

[B138] Zhang L. Qu X. Su S. Guan L. Liu P. (2012a). A novel mutation in *GJA3* associated with congenital Coppock-like cataract in a large Chinese family. Mol. Vis. 18, 2114–2118 22876138 PMC3413429

[B139] Zhang X. Wang L. Wang J. Dong B. Li Y. (2012b). Coralliform cataract caused by a novel connexin46 (*GJA3*) mutation in a Chinese family. Mol. Vis. 18, 203–210 22312188 PMC3272055

[B140] Zhou Z. Hu S. Wang B. Zhou N. Zhou S. Ma X. (2010). Mutation analysis of congenital cataract in a Chinese family identified a novel missense mutation in the connexin 46 gene (GJA3). Mol. Vis. 16, 713–719 20431721 PMC2861125

